# Complex interaction between dengue virus replication and expression of miRNA-133a

**DOI:** 10.1186/s12879-016-1364-y

**Published:** 2016-01-27

**Authors:** Jorge Andrés Castillo, Juan Camilo Castrillón, Mayra Diosa-Toro, Juan Guillermo Betancur, Georges St Laurent, Jolanda M. Smit, Silvio Urcuqui-Inchima

**Affiliations:** 1 Grupo Inmunovirología, Facultad de Medicina, Universidad de Antioquia UdeA, Calle 70 No. 52-21, Medellin, Colombia; 2 Department of Medical Microbiology, University Medical Center Groningen, University of Groningen, Groningen, The Netherlands; 3 St Laurent Institute, 317 New Boston St, Woburn, MA 01801 USA

**Keywords:** miRNA-133a, Dengue virus, Polypyrimidine tract binding protein

## Abstract

**Background:**

Dengue virus (DENV) is the most common vector-borne viral infection worldwide with approximately 390 million cases and 25,000 reported deaths each year. MicroRNAs (miRNAs) are small non-coding RNA molecules responsible for the regulation of gene expression by repressing mRNA translation or inducing mRNA degradation. Although miRNAs possess antiviral activity against many mammalian-infecting viruses, their involvement in DENV replication is poorly understood.

**Methods:**

Here, we explored the relationship between DENV and cellular microRNAs using bioinformatics tools. We overexpressed miRNA-133a in Vero cells to test its role in DENV replication and analyzed its expression using RT-qPCR. Furthermore, the expression of polypyrimidine tract binding protein (PTB), a protein involved in DENV replication, was analyzed by western blot. In addition, we profiled miRNA-133a expression in Vero cells challenged with DENV-2, using Taqman miRNA.

**Results:**

Bioinformatic analysis revealed that the 3' untranslated region (3'UTR) of the DENV genome of all four DENV serotypes is targeted by several cellular miRNAs, including miRNA-133a. We found that overexpression of synthetic miRNA-133a suppressed DENV replication. Additionally, we observed that PTB transcription , a miRNA-133a target, is down-regulated during DENV infection. Based in our results we propose that 3'UTR of DENV down-regulates endogenous expression of miRNA-133a in Vero cells during the first hours of infection.

**Conclusions:**

miRNA-133a regulates DENV replication possibly through the modulation of a host factor such as PTB. Further investigations are needed to verify whether miRNA-133a has an anti-DENV effect in vivo.

**Electronic supplementary material:**

The online version of this article (doi:10.1186/s12879-016-1364-y) contains supplementary material, which is available to authorized users.

## Background

Dengue virus (DENV) causes an estimated 390 million infections per year, making dengue the most prevalent mosquito-borne viral infection worldwide [1]. DENV belongs to the *Flaviviridae* family and four antigenically distinct virus serotypes designated 1 to 4 (DENV 1–4) have been identified to date. Infection with any of the four DENV serotypes can lead to a broad spectrum of clinical symptoms ranging from acute febrile illness to life-threatening complications such as hemorrhages and hypovolemic shock [2, 3]. Neither a vaccine nor an antiviral drug therapy exists to prevent or treat dengue diseases.

The genome of DENV consists of an 11-kilobase-long single-stranded positive sense RNA molecule, encoding one open reading frame (ORF) flanked by a 5′ untranslated region (UTR) and a 3′UTR. The viral RNA is translated as a single polyprotein that is cleaved by a combination of host cell enzymes and the viral NS2B-3 protease complex to produce three structural (C, prM/M, and E) and seven nonstructural (NS1, NS2A, NS2B, NS3, NS4A, NS4B, and NS5) proteins [4]. In addition, the flavivirus RNA produces two functional non-coding RNAs derived from the 3′UTR; the subgenomic flavivirus RNA (sfRNA) and KUN-miR-1 (reviewed in: [5]). Interestingly, Schnettler et al., (2012) demonstrated that sfRNA efficiently suppresses both the siRNA- and microRNA (miRNA)-induced RNAi pathway in mammalian and insect cells [6].

Small RNAs, such as miRNAs, are known to direct post-transcriptional regulation of gene expression [7]. MiRNAs can be derived from host or viral RNAs and can participate in a wide range of biological processes including proliferation, cell development, apoptosis and host defense [7, 8]. Host-derived miRNAs from plants, nematodes, fungi and animals have antiviral activity against many viral infections [9–11]. On the other hand, virus-derived miRNAs regulate host and/or viral gene expression in order to support viral replication [12]. The positive or negative effect of cellular or viral miRNAs on virus replication is either caused by a direct interaction of the miRNA with the genome of the virus, or by regulation of cellular factors that are important in virus replication [13–15].

Host miRNAs exhibit a variety of effects on the life cycle of DENV. For example, incorporation of the miRNA recognition element (MRE) for the hepatic-specific miR-122 in the 3′ UTR of DENV-RNA was found to suppress viral replication in transfected cells [16]. Similarly, the insertion of the MRE for the hematopoietic specific miR-142 into the DENV-2 genome restricts replication of the virus in dendritic cells and macrophages, but not in non-hematopoietic cell types [17]. In addition, experiments using a chimeric DENV/TBEV (C, prM, E from Tick-borne encephalitis virus), showed that the inclusion of the MRE for the brain-expressed miR-9 and miR-124a reduced access of the virus to the central nervous system thereby inhibiting the development of lethal encephalitis in mice [18]. Also, miR-30e* suppresses DENV replication by promoting interferon (IFN) production through the NF-κB pathway [19]. Furthermore, overexpression of Let-7c miRNA in Huh7 cells was found to decrease the infectivity of DENV [20]. Lastly, overexpression of miR-548 g-3p interferes with DENV translation and suppresses replication of all four DENV serotypes [21].

On the other hand, reports also show that miRNAs support DENV replication. For example, DENV increases the expression level of miR-146a, thereby supporting viral replication by dampening IFN production [22]. Infection with DENV also changes the miRNA-expression profile of PBMCs [23]. However, the impact of the miRNA pathway on DENV infection requires further investigation. This becomes more important if we consider that DENV encodes functional miRNAs/viral small RNAs and one of them targets specifically the virus nonstructural protein 1 gene [24]. In this study we investigated the role of miRNA-133a during DENV infection. We found that overexpression of synthetic miRNA-133a suppressed DENV replication, likely through interference with polypyrimidine tract binding protein (PTB) expression. Furthermore, our data shows that all four DENV serotypes down-regulate the expression of miRNA-133a during the first 24 h post-infection (hpi); the 3′ UTR of DENV-RNA being involved in this process.

## Methods

### Bioinformatics predictions

To identify cellular miRNAs with candidate target sites in the 3′UTR of DENV RNA, the reference genomes of all 4 serotypes were downloaded from GeneBank (Accession number: DENV-1 NC_001477; DENV-2 NC_001474; DENV-3 NC_001475; DENV-4 NC_002640) and analyzed using MicroInspector [25]. Microinspector and RNA hybrid are free algorithms available at their respective websites (ncbi.nlm.nih.gov/pubmed/15980566 and http://bibiserv.techfak.uni-bielefeld.de/rnahybrid/). MicroInspector allows the prediction of microRNA elements (MREs) for all the human miRNAs reported in the miRBase. Since the 3′UTR sequence is moderately conserved [26] and considering that a functional miRNA target site would likely be conserved among the 4 serotypes, only those common target sites to the 4 reference sequences were selected. Then, the findings of the MicroInspector algorithm were confirmed using the RNAhybrid program [27]. RNAhybrid takes into account not only the presence of a complementary sequence of the seed of the miRNA, but also the secondary structure that the miRNA-target duplex acquires when the two RNAs interact, as well as their thermodynamic stability.

### Cell lines

The mosquito C6/36 HT cell line was obtained from the ATCC and cultured as previously described [28]. Vero cells were obtained from the ATCC ( CCL-81) and grown in Dulbecco’s modified Eagle medium (DMEM) supplemented with 10 % V/V heat-inactivated fetal bovine serum (FBS), 4 mM L-glutamine, and 10 units/ml Penicillin/ 0.1 mg/ml Streptomycin (Sigma-Aldrich Chemical Co, MO, USA), at 37 °C in an atmosphere of 5 % CO_2_.

### Virus stocks and titration

Clinical isolates of DENV-1 (strain Bga-07), DENV-2 (strain 109–05) and DENV-4 (strain Bga-06) were obtained from patients with dengue hemorrhagic fever from Antioquia, Colombia (kindly provided by Dr. Díaz F.J, Grupo Inmunovirología, Facultad de Medicina, Universidad de Antioquia) and used for 3′UTR cloning. The reference strain of DENV-1, DENV-2 New Guinea C (NGC), DENV-3 and DENV-4 were provided by the Center for Disease Control (CDC, CO, USA). Viral stocks were obtained by inoculating the viruses to a monolayer of C6/36 HT cells in a 75-cm^2^ tissue culture flask with the virus at a multiplicity of infection (MOI) of 0.05 diluted in 1 ml of L-15 medium supplemented with 2 % FBS. After 3 h of adsorption, 10 ml of L15 medium supplemented with 2 % FBS were added and the cells were cultured for 5 days at 34 °C without CO_2_. The supernatant was removed from the cells and centrifuged for 5 min at 1800 rpm to pellet cellular debris, and then aliquoted for storage at −70 °C for future use. Since titration of DENV by plaque assays is time-consuming and not suitable for strains that do not plaque, virus titration was performed by flow cytometry, as previously described [29]. Briefly, the C6/36 HT cells were seeded in 12-well plates and cultured overnight at 34 °C without CO_2_. Then, they were infected with 10-fold serial dilutions of the virus and at 24 hpi, cells were harvested and resuspended in PBS. For flow cytometry analyses, the cells were fixed using a Fixation/Permeabilization buffer (eBioscience, CA, USA), centrifuged, washed twice with PBS and stained with the monoclonal antibody 4G2 (Millipore, Darmstadt, Germany). A secondary antibody, fluorescein isothiocyanate (FITC)-labeled goat anti-mouse IgG antibody (Invitrogen, Life Technologies, CA, USA) was used. Cells were analyzed on a FACScan flow cytometer using the FACSdiva software. The percentage of infected cells in each sample and the total number of cells seeded per well were used to calculate the final titer of the virus.

### Plasmid construction

The 3′ UTRs of DENV-1, −2 and −4 were amplified by PCR from viral RNA obtained from cell culture supernatants. For all 3′ UTRs, cDNA was synthesized using 200 U/μl SuperScript III RT (Thermo Scientific, NH, USA) in the presence of specific primers (forward: 5′ GAATTCG**TAG**GTGCGGCTCATTGATTGGGCTAAC 3′ that contains a stop codon (bold), and reverse: 5′ GTCGACGAACCTGTTGATTCAACAGCACC 3′). Restriction site for EcoRI and SalI (underlined) were incorporated during amplification at the 5′end of the forward and reverse primers, respectively. Transcription was conducted with 50 U/μl HotStartTaq (Thermo Scientific, NH, USA) using the same pair of primers. PCR products were purified with the QIAquick PCR Purification Kit (Qiagen, Hilden, Germany), according to the manufacturer’s recommendations, and cloned into pEGFP-C1 (Clontech, CA, USA), using the EcoRI and SalI enzymes (Thermo Scientific, NH, USA), to obtain a GFP construct bound to the 3′UTR of DENV-RNA. Cloning was verified by restriction assay and sequencing; the constructs generated are designated pGUD1, pGUD2 and pGUD4 for the DENV-1 3′UTR; DENV-2 3′UTR, and DENV-4 3′UTR, respectively. Despite our best efforts the 3′ UTR of DENV-3 could not be amplified from viral RNA from the DENV-3 isolates that we had available.

### miRNA-133a overexpression and evaluation of miRNA-133a antiviral activity

To assess the effect of miRNA-133a on DENV-2 replication, Vero cells were seeded at a density of 4x10^5^ cells/well in 12-well cell culture plates. The following day, cells were transfected with synthetic miRNA-133a mimics or with miRNA-133a antisense mimics at a final concentration of 50 nM/well (Ambion, TX, USA), using DharmaFect (Thermo Scientific, NH, USA) according to manufacturer’s instructions. At 24 h post-transfection, cells were infected with DENV-2 strain NGC at a MOI of 3 per cell, following the procedure described above. At the indicated time points, cell monolayers were harvested and the percentage of infection was measured by flow cytometry. Cell supernatants were used for viral RNA purification, and viral RNA copy number was assessed by RT-qPCR.

### Quantitation of DENV infection

At the indicated time points post-infection, Vero cells were harvested and analyzed by flow cytometry as described above. The infected cells are expressed as the percentage of infected cells over the total number of cells analyzed.

### Quantitation of viral RNA copy number

Viral RNA was extracted from culture supernatants using the QIAamp Viral RNA Mini Kit (Qiagen, Hilden, Germany), according to manufacturer’s instructions. The viral copy number was estimated by RT-qPCR using DENV-2-specific primers (forward: 5′ CAATATGCTGAAACGCGAGAGAAA 3′ and reverse: 5′CCCCATCTATTCAGAATCCCTGCT 3′),as were previously described [30]. The calculation of the genomic RNA copy number was performed based on a standard curve, as previously described [31].

### DENV infection of Vero cells and miRNA-133a expression

Vero cells were seeded at a density of 4x10^5^ cells/well in 12-well cell culture plates. The following day, cell culture medium was removed and DENV-1, DENV-3, DENV-4 (Centers for Disease Control and Prevention, CDC, GA, USA) or DENV-2 NGC, was added to give an estimated MOI of 3. Cells were incubated with the inoculum for 3 h at 37 °C with 5 % CO_2_ with gentle agitation each 30 min to allow virus adsorption. Then, medium was removed and cells were washed twice with PBS 1X, and fresh DMEM supplemented with 2 % FBS was added and incubated for 8, 16, 24, 32, 48 and 72 h at 37 °C with 5 % CO_2_. At the indicated time points, cells were harvested and total RNA was extracted using Trizol reagent (Invitrogen, CA, USA) and the expression of endogenous miRNA-133a was assessed by quantitative RT-PCR (RT-qPCR).

### pGUD1, pGUD2 and pGUD4 transient transfection

Vero cells were seeded at a density of 2x10^5^ cells/well in 24-well plates and immediately transfected using 2 μg of pGUD1, pGUD2 and pGUD4 and 2 μl of Lipofectamine 2000 reagent (Invitrogen, CA, USA), according to manufacturer’s instruction. After 5 h, the transfection medium was removed and replaced by fresh DMEM supplemented with 2 % FBS and incubated for 12, 24, 48 and 72 additional hours. At the indicated time points, cells were harvested and total RNA extraction was performed using the Trizol reagent (Invitrogen, CA, USA) following manufacturer’s instructions. The RNA concentration was measured using a NanoDrop spectrophotometer (Nano Drop Technologies, CA, USA). Expression of endogenous miRNA-133a was determined using a Taqman miRNA assay (Applied Biosystems, CA, USA). Reverse transcription was carried out using 10 ng RNA to produce cDNA. RT-qPCR reactions were performed in triplicates with each cDNA template on the Bio-Rad CFX96 real-time Detection System (Bio-Rad, CA, USA), using the SYBR green Master Mix (Thermo Scientific). Ct (Cycle-threshold) values were calculated for each reaction and normalized to an uninfected control and to the 18S RNA (ΔΔCt) to obtain the fold change. The transfection efficiency was verified 24 h post-transfection (hpt) by assessing the expression of GFP by fluorescence microscopy.

### miRNA-133a and DENV infection alter the expression of PTB mRNA

To assess the effect of miRNA-133a on PTB mRNA expression, Vero cells were seeded at a density of 8x10^5^ cells/well in 6-well cell culture plates. The following day, cells were transfected with 50 or 100 μM synthetic miRNA-133a mimics or with the miRNA-133a mimic inhibitor (Ambion, TX, USA). To determine the effect of DENV-2 on PTB expression, Vero cells were challenged with DENV-2 and the expression of PTB was evaluated 12, 24 and 48 hpi by western blot. Alternatively, Vero cells were transfected with synthetic miRNA-133a mimics and challenged with DENV-2 24 h later. Then PTB expression level was determined by western blot at 12, 24 and 48 h later.

### Western blot analysis

At the respective times, cells were lysed with a lysis solution (Applied Biosystems, CA, USA) and the protein concentration was determined using a BCA Protein Assay kit (Pierce, Thermo scientific, NH, USA). Equal amounts of sample lysate were separated using 10 % SDS-PAGE and transferred onto a nitrocellulose membrane. A primary monoclonal antibody against PTB (Invitrogen, CA, USA) or GFP (Roche) and a secondary anti-mouse IgG antibody conjugated with horseradish peroxidase (HRP) (Santa Cruz Biotechnology, CA, USA) were used. Finally, signals were detected using the chemiluminescence ECL™ detection system (Pierce).

### Statistical analysis

For all assays three independent experiments were carried out and the data are presented as median with the range. Statistical significance was determined with the two-way Anova test with a confidence interval of 95 %.

## Results

### 3′ UTR of DENV-1 to −4 contains potential cellular miRNAs binding sites

**Fig. 1:**
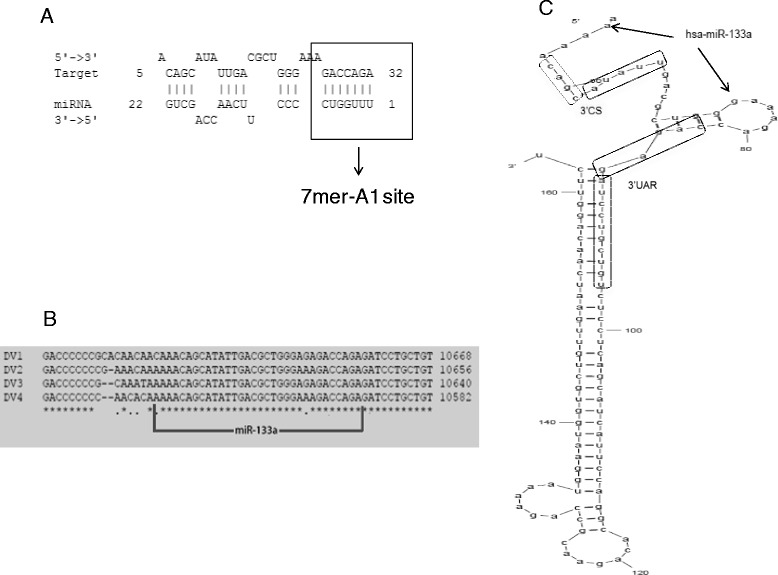
Human miRNA-133a is predicted to target the 3'UTR of DENV. Using sequence alignment and Microinspector analysis, a miRNA-133a target sequence was predicted within the DENV 3'UTR sequence, which fulfills the seed sequence rule (perfect hybridization of bases 2–8 or 2–9 from the 5' end of the miRNA with the target RNA) plus binding in a 7mer-A1 fashion (**a**). The predicted target sequence is present in all four DENV serotypes (**b**). The location of the target sequence is in a loop of the 3'UTR known as the 3'SL, that contains the elements known as 3'CS and 3'UAR (**c**)

As reported previously, human miRNAs can target viral genomes [13, 14, 32]. Therefore, we first sought to investigate whether the 3’UTR of DENV RNA contains potential miRNA binding sites, using MicroInspector software [25]. The sequences of the 3′UTRs plus 374 nucleotides of the coding region of NS5 from all four dengue serotypes were aligned. A total of 108 miRNAs for DENV-1, 80 for DENV-2, 94 for DENV-3 and 89 for DENV-4 were predicted to target the 3′UTR (Additional file 1). Since the 3′UTR sequence is moderately conserved [26] and with the hypothesis that a functional miRNA target site would be conserved among all four DENV serotypes, only those target sites common to the 4 reference sequences were selected. In total, 13 miRNAs fulfilled these criteria (Table 1). Interestingly, most of the target sites localized to a single region, highlighting a “hotspot” of potential MREs (Fig. 1). The findings of the MicroInspector algorithm were then confirmed using the RNAhybrid program [27]. RNAhybrid considers the presence of a complementary sequence of the seed of the miRNA, the secondary structure that the miRNA-target duplex acquires when the two RNAs interact, as well as their thermodynamic stability.

**Table 1:** miRNAs predicted to target a conserved region of DENV-1 to 4 3'UTR

miRNA	DENV1	DENV2	DENV3	DENV4
miR-let-7a-2-star	10645, 10465	10451	10438	10374
miR-let-7c	10640	10628	10612	10554
miR-1254	10659	10647	10631	10573
miR-1290	10632	10620	10604	10546
miR-133a	10626	10614	10598	10540
miR-16	10689	10677	10661	10603, 10606
miR-199a-5p	10616	10547	10588	10530
miR-199b-5p	10616	10604	10588	10530
miR-330-5p	10569, 10628	10616	10600	10542, 10485
miR-484	10633	10621	10605	10547
miR-744	10590, 10634	10623	10531, 10562, 10607	10549, 10318
miR-769-5p	10641	10629	10613	10555
miR-9	10628	10616	10600	10542

The bioinformatic analysis with RNAhybrid predicted a miRNA-133a target sequence in the “hotspot” of the 3′UTR (Fig. 1a). The interaction between the miRNA and the target RNA fulfills the seed sequence rule: perfect hybridization of bases 2–8 or 2–9 from the 5′ end of the miRNA known as 7mer-A1, with the target RNA [7]. Consistently, our bioinformatic analysis predicted a miRNA-133a binding site in the 3′UTRs of all four serotypes of DENV (Fig. 1b). Specifically, the target sequence localizes to a loop of the 3′UTR known as the 3′SL, which contains the elements known as 3′CS (nt 10620 to 10628) and 3′UAR (nt 10642 to 10657; Fig. 1c), both required for genome circularization and viral viability. Interestingly, miRNA-133a has been reported to target the PTB mRNA (Additional file 2) [33, 34], a cellular protein that binds to the 3′ and 5′UTR of the ssRNA of DENV and is required for viral replication and translation [35–37].

**Fig. 2:**
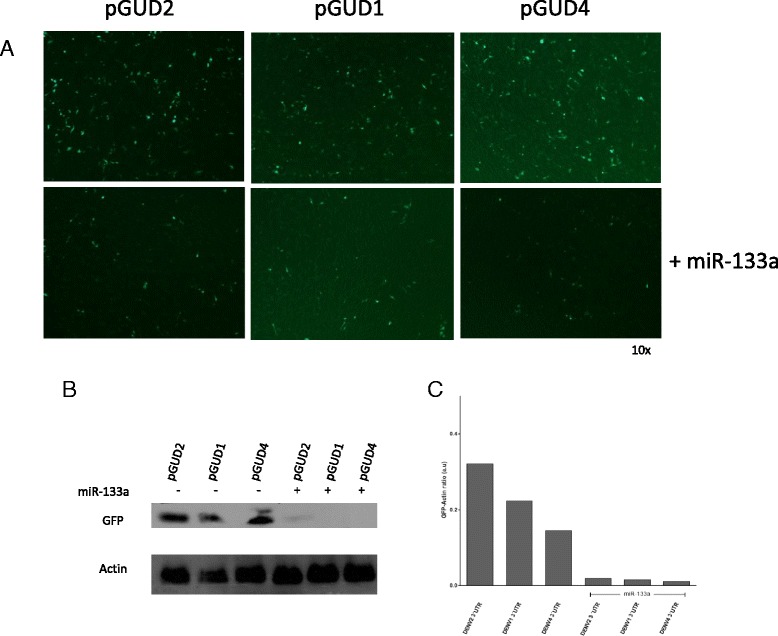
MiRNA-133a alters the expression of GFP-fused to the DENV RNA 3'UTR. Vero cells were co-transfected with pGUD1, pGUD2, pGUD4 or synthetic miRNA-133a mimics and 24 h later, the expression of GFP was assessed by fluorescence microscopy (**a**) and western blot (**b**). Actin was used as a loading control. Semi-quantitative data of the western blots were assessed using Image J (**c**). A representative experiment is shown. In total three experiments were performed

To further determine whether miRNA-133a has a functional effect on these putative binding sites, and if indeed this miRNA interacts with the 3′UTR of DENV RNA, we inserted the 3′UTR sequence of DENV1, DENV-2 or DENV-4 RNAs into the 3′ terminus of the GFP reporter gene in pEGFP-C1. Each construct (pGUD1, pGUD2 and pGUD4) was used for co-transfection with the synthetic miRNA-133a mimic in Vero cells and GFP expression was determined (Fig. 2). The results showed that miRNA-133a decreased the expression of GFP as determined by fluorescence microscopy (Fig. 2a) and western blot analysis (Fig. 2b and c). These results are consistent with our bioinformatic analysis and suggest that miRNA-133a interacts with the 3′UTR of all four DENV serotypes.

### miRNA-133a overexpression suppresses DENV-2 replication

**Fig. 3:**
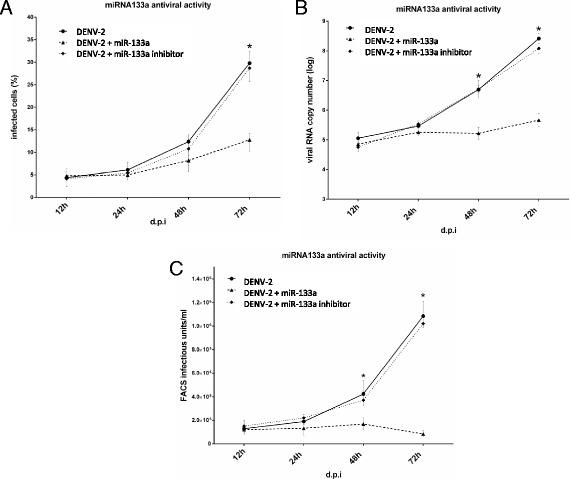
miRNA-133a suppresses DENV-2 replication. Vero cells were transfected with the synthetic form of miRNA-133a or with the miRNA-133a inhibitor as mimic, both at a final concentration of 50 μM. At 24 hpt, cells were challenged with DENV-2 at a MOI of 3 and infection was evaluated at 12, 24, 48 and 72 hpi. **a** Flow cytometry of DENV-2 challenged cells, miRNA-133a transfected and infected cells, and miRNA inhibitor transfected and infected cells. **b** Viral RNA copy number of DENV-2 infected cells, miRNA-133a transfected and infected cells, and miRNA inhibitor transfected and infected cells assessed by RT-PCR in culture supernatants. **c** Viral titer of DENV-2 infected cells, miRNA-133a transfected and infected cells, and miRNA inhibitor transfected and infected cells assessed by flow cytometry. Data are shown as median ± range from three repeated experiments. (*) Statistically significant difference compared to the control (*p* < 0.05)

Next, we determined if miRNA-133a modulates DENV infection in Vero cells. Synthetic miRNA-133a or inhibitors of miRNA-133a were transfected in Vero cells and at 24 hpi cells were challenged with DENV-2 at a MOI of 3. Viral RNA copies were quantified by RT-qPCR and the percentage of infected cells was determined by flow cytometry (using the 4G2 antibody), at 12, 24, 48 and 72 hpi . As shown in Fig. 3a, during the first 24 hpi, overexpression of miRNA-133a did not influence the percentage of infected cells. The percentage of infected cells was however lower at 48 hpi, and reached significance at 72 hpi. At 72 hpi, the number of infected cells was 80 % reduced when compared to the positive control. When Vero cells were challenged with DENV-2 in the presence of inhibitors of miRNA-133a, we found no difference compared to the DENV-2-infected cells. To confirm that miRNA-133a negatively influences DENV replication, viral RNA copies were quantified in the cell culture supernatants (Fig. 3b). A 3 log reduction in viral RNA copy number was seen in cells overexpressing miRNA-133a at 72 hpi. We also observed a 10-fold reduction in viral titer at 72 hpi, using flow cytometry (Fig. 3c). Taken together, our results indicate that overexpression of host miRNA-133a potently suppresses DENV-2 replication.

### DENV-2 infection up-regulates the expression of PTB at early stages of infection; miRNA-133a represses PTB

**Fig. 4:**
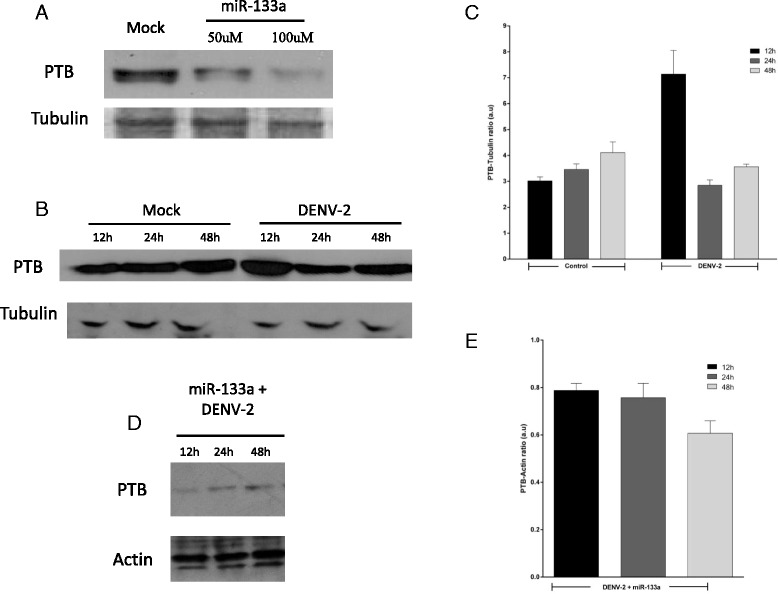
DENV up-regulates the expression of PTB through negative regulation of miRNA-133a. Vero cells were transfected with the synthetic form of miRNA-133a at a final concentration of 50 μM or 100 μM, and 24 h post-transfection PTB expression was assessed by Western blotting (**a**). Vero cells were challenged with DENV-2 NGC with a MOI of 3. PTB expression was measured by Western blotting at 12, 24 and 48 hpi (**b**). Finally, Vero cells were transfected with the synthetic form of miRNA-133a at a final concentration of 100 μM, and 24 hpt were challenged with DENV-2 at a MOI of 3. PTB expression was then measured by western blotting at 12, 24, 48 and 72 h (**d**). For (**a**, **b**), tubulin was used as loading control; for (**d**), actin was used as loading control. Semi-quantitative data of the western blot was assessed using Image J (**c**, **e**)

Since the PTB mRNA contains the miRNA-133a target sequence [34, 38], we evaluated the expression level of PTB in Vero cells with and without prior transfection of miRNA-133a by western blot. As shown in Fig. 4a, overexpression of synthetic miRNA-133a suppressed PTB expression.

It was previously reported that in infected cells, PTB moves from the nucleus to the cytoplasm and contributes to DENV replication [35, 37]. Thus, we evaluated whether DENV infection alters the level of expression of this protein. According to previous reports increased PTB expression was observed in DENV-2 infected Vero cells at 12 hpi, compared to uninfected cells (Fig. 4b). Based on this result and since it is known that PTB plays a vital role during DENV replication, we examined if PTB expression is down-regulated in DENV-infected Vero cells overexpressing miRNA-133a. We found a slight decrease in the level of PTB expression at 12 hpi (Fig. 4d and e), but the expression was slightly increased at 24 and 48 hpi. Taken together, our results suggest a possible relationship between DENV infection, miRNA-133a, and expression of PTB.

### MiRNA-133a expression is down-regulated by DENV-1 to −4 at early stages of infection

**Fig. 5:**
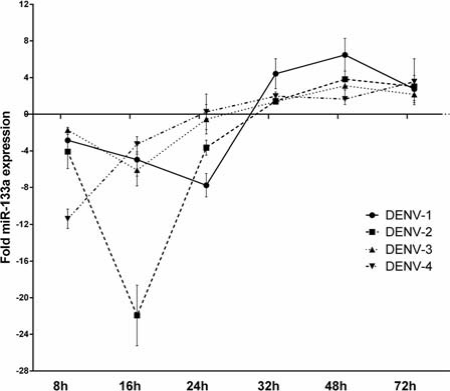
DENV infection induces down-regulation of miRNA-133a. Vero cells were infected with DENV-2 at a MOI of 3. miRNA-133a expression was evaluated at 8, 16, 24, 32, 48, 72 hpi by RT-PCR and normalized to an uninfected control and to the 18S RNA (ΔΔ Ct). Data are shown as median ± range from three repeated experiments

To investigate if Vero cells express miRNA-133 we next determined the endogenous expression (CT) level of miRNA-133a in Vero cells by RT-qPCR. The results show that Vero cells indeed express miRNA-133a (Additional file 3). Therefore, we next aimed to investigate if miRNA-133a is regulated during DENV infection. To this end we challenged Vero cells with DENV-1, −2, −3 or −4 at a MOI of 3 per cell and quantified the endogenous expression of miRNA-133a at 8, 16, 24, 32, 48 and 72 hpi by RT-qPCR. To analyze the effect of DENV infection on miRNA-133a expression, we normalized the fold-change of miRNA-133a in Vero cells challenged with DENV to uninfected Vero cells and to 18S RNA (ΔΔCt; Additional file 3). Although the extent of down-regulation for each serotype varied, the level of expression of miR-133a decreased in infected cells during the first 8 hpi (Fig. 5). Repression of miRNA-133a expression intensified at 16 hpi for DENV-2 and continued for DENV-1 till 24 h, whereas in the case of DENV-4 and DENV-3 infection, the level of miRNA-133a increased back to the basal level. The largest change was observed at 16 hpi for all serotypes, highlighting a 22-fold decrease induced by DENV-2. At later time points, the expression of miRNA-133a recovered and up-regulation occurred in the presence of all DENV serotypes (Fig. 5). These results show that all four DENV serotypes down-regulate the expression of miRNA-133a early in infection, suggesting that this miRNA may play a role in the antiviral response.

### The 3′UTR of DENV inhibits the expression of miRNA-133a

**Fig. 6:**
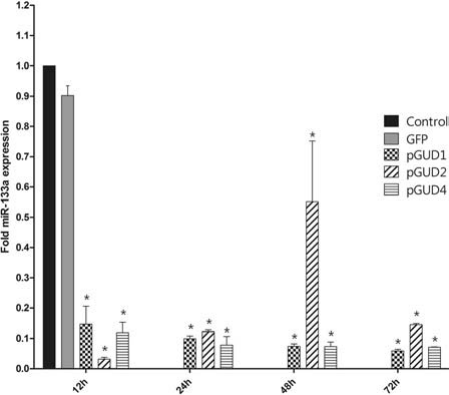
The expression of miRNA-133a is regulated by the 3'UTR of DENV. Vero cells were transfected with a pEGFP plasmid carrying the 3'UTR of DENV-1, −2, or −4. miRNA-133a expression was evaluated at 12, 24, 48 and 72 hpt by RT-PCR and normalized to an untransfected control and to the 18S RNA (ΔΔ Ct). Data are shown as median ± range from three repeated experiments. (*) Statistically significant difference compared to the control (*p* < 0.05)

It has been reported that sfRNA, derived from the 3′UTR, is abundantly expressed during flavivirus infection in cultured cells [39]. It was described to function as an RNAi suppressor during virus replication through interference with the processing of the dsRNA template by Dicer [6]. Consequently, the involvement of the 3′UTR of DENV RNA in down-regulation of miRNA-133a expression was investigated. The 3′UTR of DENV-1, −2 and 4 was cloned into the pEGFP-C1 plasmid and the constructs obtained were used to transfect Vero cells. As controls, the empty pEGFP-C1 vector, as well as non-transfected cells were used. Expression of miRNA-133a was evaluated at 12, 24, 48 and 72 h post-transfection (hpt) by RT-qPCR and the expression was normalized to the non-transfected control cells and to 18S RNA . As shown in Fig. 6, the 3′UTR of DENV-1, −2, and −4 induced a strong decrease of more than 6-fold in the expression of miRNA-133a at all the time points examined, with the exception of the 3′UTR of DENV-2 at 48 hpt. The 3′UTR of DENV-2 induced the highest down regulation with a 30-fold change at 12 hpt (Fig. 6).

## Discussion

Cellular miRNAs participate in the life cycles of many viruses [13, 14, 32], including DENV [40]. In this study we demonstrate that overexpression of miRNA-133a impairs DENV-2 replication, affecting both the percentage of infected cells and the number of produced viral RNA copies. Although it is not clear how miRNA-133a alters DENV replication we propose that, based on our bioinformatic screening, miRNA133a directly binds to a sequence localized in the 3′SL loop of the 3′UTR. This loop contains the 3′CS and 3′UAR elements that are required for genome circularization and viral viability [41–43]. This hypothesis is strengthened by the results obtained with the 3′UTR-GFP DENV plasmids. Further studies are needed to confirm this hypothesis, as well as to determine which mechanisms are used by miRNA-133a to regulate DENV infection.

Interestingly, miRNA-548 g-3p was recently reported to bind to the stem loop A (SLA) promoter in the DENV 5′UTR, a very important element involved in DENV replication, and inhibits DENV replication [21]. Both, DENV 3′UTR and 5′UTR contain sequences that are implicated in translation, replication and cyclisation. Furthermore, the UTR of DENV interacts with cellular proteins, including PTB [36, 44]. PTB has been implicated in multiple steps of pre-mRNA processing, polyadenylation regulation, and viral/cellular IRES-dependent translation of RNA [45–50]. In Vero cells, silencing of PTB expression alters both virus translation and replication whereas overexpression of PTB increases DENV propagation [35]. PTB binds specifically to the conserved sequence 1 and long stem-loop structures of the 3′UTR of DENV [44] and promotes viral RNA replication, possibly by acting as a RNA helicase [35, 37, 44]. Here we observed that PTB expression is increased in Vero cells challenged with DENV, with a peak of expression at 12 hpi, indicating that the regulation occurs early in infection. Interestingly, DENV-infected Vero cells overexpressing miRNA-133a clearly showed a marked reduction in PTB expression at 12 hpi, whereas at later times (24, 48 hpi) a slight increase expression was observed. Therefore we hypothesize that PTB is crucial in the first hours of viral replication. The virus may induce down-regulation of miRNA-133a in order to maintain high levels of PTB early in infection thereby facilitating viral replication. Based on these results and since previous reports show that miRNA-133a inhibits PTB mRNA translation by binding to the 3-UTR of the PTB mRNA [33, 34], we suggest that lower levels of PTB alter the circularization of DENV RNA, an essential step for DENV RNA translation and RNA synthesis. Our hypothesis is that miRNA-133a acts as an anti-DENV agent and this is supported by the fact that all four serotypes of DENV are able to decrease the endogenous expression of miRNA-133a in Vero cells early in infection. We did not observe cell death, however there is a possibility that overexpression of miRNA-133a has cytotoxic effects and the observed antiviral activity may be due to an indirect phenomenon. The biological consequence of such changes in miRNA-133a levels is unknown, but we propose that it can induce PTB expression, and in turn promote DENV replication, as previously reported [44]. In fact, PTB depletion and PTB inhibition was previously reported to reduce DENV replication, suggesting an important role for this protein in the DENV life cycle [37].

Several studies described the pathway by which DENV down-regulates the expression of cellular miRNAs. For example, NS4B was described to act as a potent RNAi suppressor [51]. Furthermore, for West Nile virus, sfRNA was described to suppress the siRNA- and miRNA-induced RNAi pathway [6]. Our results suggest a role for the 3′UTR of DENV RNA in the regulation of cellular miRNA expression. Although this is a novel report showing a link between DENV infection and host miRNA*,* there is a growing number of studies on the function of the miRNA/RNAi machinery in DENV replication [52–54]. These data support the idea that noncoding sequences of DENV such as the 3′UTR might be involved in miRNA synthesis, as we report here. Furthermore, sfRNA derived from the Flavivirus 3′UTR is involved in inhibiting the antiviral activity of type II IFN and in suppressing RNAi activity in insect and mammalian cells [55].

Several viral factors have been described that can inhibit the RNAi machinery. The HIV-1 Nef, an accessory protein, interacts with Ago2 protein and function as a suppressor of RNAi [56]. Also, the HIV-1 proteins Tat and Rev can suppress Dicer-dependent RNA silencing through RNA binding proteins that contain arginine-rich motifs (the short arginine-rich linear motif of the HIV-1 regulatory proteins inhibits Dicer-dependent RNA interference). For DENV, the NS4B protein has RNAi suppressor activity [51]. These data together with data from others [6], show that mammalian viruses, similar to insect and plant viruses, encode for proteins or RNA sequences with RNAi silencing suppression (RSS) activity. Our data also suggest that an RSS role for the 3′UTR of DENV will be assigned. Although the data of our study can lead to understanding further the function of miRNA-133a in DENV replication, further studies are needed to better understand the biological significance of our results and their application as an antiviral strategy. Particularly, the study of miRNA-133a expression changes in response to DENV infection in DENV targets cells could provide very interesting clues about the host factors that are involved during DENV infection.

## Conclusions

In conclusion, we report for the first time the involvement of miRNA-133a in modulating DENV-2 replication, possibly by altering the expression of host factors such as PTB. In addition we found that infection of Vero cells with each of the 4 DENV serotypes or plasmid construct encoding the 3′UTR of DENV RNA transfection resulted in decreases expression of miRNA-133a.

## Additional files

Additional file 1: **MiRNAs predicted to target DENV-1 to −4 3′UTR region.** (PDF 194 kb)

Additional file 2: **miRNA-133a predicted targets in the PTB1 gene. Predicted targets of MyomiRs are shown with the positions of target sequences in the 3′ UTR of mammalian mRNAs.** (PDF 126 kb)

Additional file 3: **Endogenous expression of miR-133a in Vero cells infected with DENV-2.** (DOCX 14 kb)
