# Supplementary material for: Complex interaction between dengue virus replication and expression of miRNA-133a
Source: BMC Infect Dis. 2016 Jan 27;16:29. doi: 10.1186/s12879-016-1364-y (PMC4728791; doi:10.1186/s12879-016-1364-y)
Supplement: Supplementary file 1 — MiRNAs predicted to target DENV-1 to −4 3′UTR region. (PDF 194 kb) [file 12879_2016_1364_MOESM1_ESM.pdf]

Serotype (code)	DV1 (NC_001477.1)		
UTR starts at (nt)	10274		
	Position in the genome	Position in the 3'UTR	Name
	10645	371	hsa-miR-637
	10337	63	hsa-miR-34c-5p
	10423	149	hsa-miR-1266
	10414	140	hsa-miR-27a-star
	10659	385	hsa-miR-1254
	10496	222	hsa-miR-642
	10338	64	hsa-miR-34b-star
	10490	216	hsa-miR-645
	10689	415	hsa-miR-16
	10576	302	hsa-miR-146a-star
	10423	149	hsa-miR-1915
	10471	197	hsa-miR-202-star
	10628	354	hsa-miR-9
	10626	352	hsa-miR-133a
	10632	358	hsa-miR-1290
	10583	309	hsa-miR-615-5p
	10584	310	hsa-miR-615-5p
	10585	311	hsa-miR-615-5p
	10548	274	hsa-miR-1301
	10447	173	hsa-miR-1301
	10686	412	hsa-miR-1179
	10549	275	hsa-miR-143-star
	10342	68	hsa-miR-27a
	10423	149	hsa-miR-1471
	10525	251	hsa-miR-1471
	10657	383	hsa-miR-378
	10442	168	hsa-miR-1237
	10414	140	hsa-miR-27b-star
	10673	399	hsa-miR-129-5p
	10674	400	hsa-miR-129-5p
	10347	73	hsa-miR-193b-star
	10659	385	hsa-miR-146b-3p
	10350	76	hsa-miR-1908
	10641	367	hsa-miR-769-5p
	10473	199	hsa-miR-508-5p
	10616	342	hsa-miR-199a-5p
	10393	119	hsa-miR-1287
	10540	266	hsa-miR-623
	10541	267	hsa-miR-623
	10336	62	hsa-miR-657
	10336	62	hsa-miR-103
	10343	69	hsa-miR-600
	10337	63	hsa-miR-449b
	10691	417	hsa-miR-421
	10568	294	hsa-miR-522-star
	10546	272	hsa-miR-622
	10568	294	hsa-miR-518e-star
	10568	294	hsa-miR-518f-star
	10539	265	hsa-miR-1914
	10518	244	hsa-miR-191-star
	10591	317	hsa-miR-147b
	10633	359	hsa-miR-484
	10563	289	hsa-miR-502-5p
	10356	82	hsa-miR-502-5p
	10335	61	hsa-miR-16-1-star
	10337	63	hsa-miR-34a
	10689	415	hsa-miR-29b
	10398	124	hsa-miR-1538
	10591	317	hsa-miR-147
	10539	265	hsa-miR-518c-star
	10347	73	hsa-miR-886-5p
	10506	232	hsa-miR-30c-1-star
	10645	371	hsa-let-7a-2-star
	10465	191	hsa-let-7a-2-star
	10455	181	hsa-miR-132-star
	10465	191	hsa-let-7g-star
	10475	201	hsa-miR-138-2-star
	10646	372	hsa-miR-612
	10385	111	hsa-miR-1204
	10392	118	hsa-miR-1307
	10616	342	hsa-miR-1307
	10483	209	hsa-miR-554
	10353	79	hsa-miR-422a
	10425	151	hsa-miR-1274a
	10632	358	hsa-miR-609
	10489	215	hsa-miR-328
	10569	295	hsa-miR-330-5p
	10628	354	hsa-miR-330-5p
	10704	430	hsa-miR-492
	10541	267	hsa-miR-211
	10398	124	hsa-miR-29b-2-star
	10579	305	hsa-miR-1238
	10542	268	hsa-miR-1238
	10495	221	hsa-miR-1238
	10495	221	hsa-miR-150
	10311	37	hsa-miR-1978
	10478	204	hsa-miR-93-star
	10616	342	hsa-miR-199b-5p
	10308	34	hsa-miR-640
	10478	204	hsa-miR-149
	10490	216	hsa-miR-149
	10564	290	hsa-miR-22-star
	10418	144	hsa-miR-1226-star
	10437	163	hsa-miR-1225-5p
	10551	277	hsa-miR-555
	10640	366	hsa-let-7c
	10658	384	hsa-miR-1299
	10459	185	hsa-miR-103-2-star
	10568	294	hsa-miR-523-star
	10490	216	hsa-miR-345
	10418	144	hsa-miR-654-5p
	10442	168	hsa-miR-331-3p
	10579	305	hsa-miR-877-star
	10551	277	hsa-miR-196a
	10338	64	hsa-miR-196a
	10336	62	hsa-miR-107
	10437	163	hsa-miR-125b-1-sta
	10352	78	hsa-miR-1228-star
	10551	277	hsa-miR-196b
	10568	294	hsa-miR-519b-5p
	10388	114	hsa-miR-219-2-3p
	10686	412	hsa-miR-371-3p
	10633	359	hsa-miR-296-3p
	10568	294	hsa-miR-519c-5p
	10436	162	hsa-miR-23a-star
	10435	161	hsa-miR-23a-star
	10480	206	hsa-miR-346
	10702	428	hsa-miR-649
	10569	295	hsa-miR-326
	10471	197	hsa-miR-662
	10568	294	hsa-miR-519a-star
	10590	316	hsa-miR-744
	10634	360	hsa-miR-744
	10315	41	hsa-miR-584
	Total number of miRNAs*:		108

DV2 (NC_001474.2)		
10273		
Position in the genome	Position in the 3'UTR	Name
10634	361	hsa-miR-637
10523	250	hsa-miR-214-star
10647	374	hsa-miR-1254
10677	404	hsa-miR-16
10565	292	hsa-miR-146a-star
10490	217	hsa-miR-149-star
10549	276	hsa-miR-766
10398	125	hsa-miR-650
10616	343	hsa-miR-9
10538	265	hsa-miR-1184
10614	341	hsa-miR-133a
10620	347	hsa-miR-1290
10575	302	hsa-miR-615-5p
10537	264	hsa-miR-1301
10449	176	hsa-miR-1301
10518	245	hsa-miR-193a-5p
10390	117	hsa-miR-604
10674	401	hsa-miR-1179
10319	46	hsa-miR-1909
10623	350	hsa-miR-1909
10321	48	hsa-miR-27a
10489	216	hsa-miR-1914-star
10464	191	hsa-miR-181a-2-sta
10541	268	hsa-miR-185-star
10645	372	hsa-miR-378
10568	295	hsa-miR-1237
10662	389	hsa-miR-129-5p
10647	374	hsa-miR-146b-3p
10545	272	hsa-miR-1908
10629	356	hsa-miR-769-5p
10440	167	hsa-miR-1308
10547	274	hsa-miR-199a-5p
10604	331	hsa-miR-199a-5p
10679	406	hsa-miR-421
10302	29	hsa-miR-2113
10535	262	hsa-miR-1207-3p
10621	348	hsa-miR-484
10526	253	hsa-miR-720
10468	195	hsa-miR-1269
10518	245	hsa-miR-296-5p
10517	244	hsa-miR-296-5p
10335	62	hsa-miR-502-5p
10411	138	hsa-miR-141-star
10491	218	hsa-miR-939
10461	188	hsa-miR-18a-star
10326	53	hsa-miR-608
10325	52	hsa-miR-608
10550	277	hsa-miR-30a-star
10633	360	hsa-miR-30c-1-star
10633	360	hsa-miR-1224-5p
10451	178	hsa-let-7a-2-star
10414	141	hsa-miR-500-star
10334	61	hsa-miR-432
10300	27	hsa-miR-132-star
10441	168	hsa-miR-132-star
10321	48	hsa-miR-27b
10376	103	hsa-miR-1307
10556	283	hsa-miR-554
10332	59	hsa-miR-422a
10568	295	hsa-miR-942
10531	258	hsa-miR-942
10532	259	hsa-miR-412
10465	192	hsa-miR-572
10616	343	hsa-miR-330-5p
10692	419	hsa-miR-492
10531	258	hsa-miR-1238
10568	295	hsa-miR-150
10604	331	hsa-miR-199b-5p
10553	280	hsa-miR-22-star
10351	78	hsa-miR-29a-star
10278	5	hsa-miR-29a-star
10468	195	hsa-miR-150-star
10628	355	hsa-let-7c
10281	8	hsa-miR-29c-star
10646	373	hsa-miR-1299
10356	83	hsa-miR-663b
10478	205	hsa-miR-1251
10550	277	hsa-miR-30e-star
10403	130	hsa-miR-194-star
10546	273	hsa-miR-194-star
10462	189	hsa-miR-892a
10568	295	hsa-miR-877-star
10478	205	hsa-miR-302d-star
10674	401	hsa-miR-371-3p
10690	417	hsa-miR-649
10565	292	hsa-miR-326
10558	285	hsa-miR-326
10538	265	hsa-miR-1205
10623	350	hsa-miR-744
10294	21	hsa-miR-584
		80

DV3 (NC_001475.2)		
10268		
Position in the genome	Position in the 3'UTR	Name
10422	154	hsa-miR-557
10618	350	hsa-miR-637
10307	39	hsa-miR-34c-5p
10631	363	hsa-miR-1254
10515	247	hsa-miR-642
10463	195	hsa-miR-645
10661	393	hsa-miR-16
10530	262	hsa-miR-149-star
10293	25	hsa-miR-1180
10411	143	hsa-miR-1915
10412	144	hsa-miR-1915
10600	332	hsa-miR-9
10598	330	hsa-miR-133a
10604	336	hsa-miR-1290
10526	258	hsa-miR-615-5p
10449	181	hsa-miR-221
10658	390	hsa-miR-1179
10437	169	hsa-miR-143-star
10357	89	hsa-miR-647
10518	250	hsa-miR-516a-3p
10474	206	hsa-miR-1914-star
10475	207	hsa-miR-1914-star
10476	208	hsa-miR-1914-star
10350	82	hsa-miR-185-star
10349	81	hsa-miR-185-star
10629	361	hsa-miR-378
10415	147	hsa-miR-1237
10514	246	hsa-miR-1237
10646	378	hsa-miR-129-5p
10518	250	hsa-miR-516b-star
10450	182	hsa-miR-1178
10631	363	hsa-miR-146b-3p
10391	123	hsa-miR-658
10392	124	hsa-miR-1908
10613	345	hsa-miR-769-5p
10427	159	hsa-miR-128
10320	52	hsa-miR-663
10588	320	hsa-miR-199a-5p
10515	247	hsa-miR-623
10424	156	hsa-miR-33b-star
10307	39	hsa-miR-449b
10366	98	hsa-miR-302a-star
10663	395	hsa-miR-421
10366	98	hsa-miR-371-5p
10605	337	hsa-miR-484
10511	243	hsa-miR-720
10545	277	hsa-miR-617
10326	58	hsa-miR-502-5p
10353	85	hsa-miR-635
10354	86	hsa-miR-635
10552	284	hsa-miR-378-star
10447	179	hsa-miR-18a-star
10441	173	hsa-miR-147
10285	17	hsa-miR-30b-star
10366	98	hsa-miR-498
10317	49	hsa-miR-886-5p
10617	349	hsa-miR-30c-1-star
10478	210	hsa-miR-30c-1-star
10617	349	hsa-miR-1224-5p
10526	258	hsa-let-7g
10438	170	hsa-let-7a-2-star
10438	170	hsa-let-7g-star
10618	350	hsa-miR-612
10508	240	hsa-miR-636
10505	237	hsa-miR-1307
10323	55	hsa-miR-422a
10517	249	hsa-miR-942
10553	285	hsa-miR-942
10428	160	hsa-miR-23b
10399	131	hsa-miR-1274a
10604	336	hsa-miR-609
10292	24	hsa-miR-572
10268	0	hsa-miR-328
10268	0	hsa-miR-328
10462	194	hsa-miR-328
10600	332	hsa-miR-330-5p
10676	408	hsa-miR-492
10515	247	hsa-miR-211
10283	15	hsa-miR-29b-2-star
10455	187	hsa-miR-29b-2-star
10468	200	hsa-miR-1238
10468	200	hsa-miR-150
10472	204	hsa-miR-23b-star
10459	191	hsa-miR-93-star
10588	320	hsa-miR-199b-5p
10392	124	hsa-miR-1226-star
10274	6	hsa-miR-550
10345	77	hsa-miR-29a-star
10525	257	hsa-miR-555
10612	344	hsa-let-7c
10630	362	hsa-miR-1299
10524	256	hsa-miR-675
10456	188	hsa-miR-29b-1-star
10465	197	hsa-miR-1251
10399	131	hsa-miR-331-3p
10510	242	hsa-miR-194-star
10322	54	hsa-miR-1228-star
10377	109	hsa-miR-564
10658	390	hsa-miR-371-3p
10605	337	hsa-miR-296-3p
10409	141	hsa-miR-23a-star
10408	140	hsa-miR-23a-star
10459	191	hsa-miR-639
10674	406	hsa-miR-649
10418	150	hsa-miR-1234
10531	263	hsa-miR-744
10562	294	hsa-miR-744
10607	339	hsa-miR-744
		94

DV4 (NC_002640.1)			
10266			
Position in the genome	Position in the 3'UTR	Name	
10277	11	hsa-miR-140-5p	
10484	218	hsa-miR-518d-5p	
10471	205	hsa-miR-665	
10346	80	hsa-miR-637	*Different possition in DENV4
10444	178	hsa-miR-27a-star	
10573	307	hsa-miR-1254	
10405	139	hsa-miR-642	
10603	337	hsa-miR-16	
10606	340	hsa-miR-16	
10492	226	hsa-miR-146a-star	
10413	147	hsa-miR-149-star	
10532	266	hsa-miR-1180	
10533	267	hsa-miR-1180	
10376	110	hsa-miR-187	
10293	27	hsa-miR-373-star	
10386	120	hsa-miR-222-star	
10542	276	hsa-miR-9	
10540	274	hsa-miR-133a	
10546	280	hsa-miR-1290	
10500	234	hsa-miR-615-5p	*Different possition
10343	77	hsa-miR-615-5p	
10499	233	hsa-miR-615-5p	
10502	236	hsa-miR-615-5p	
10342	76	hsa-miR-615-5p	
10464	198	hsa-miR-1301	
10549	283	hsa-miR-1909	
10410	144	hsa-miR-1914-star	
10412	146	hsa-miR-1914-star	
10411	145	hsa-miR-1914-star	
10273	7	hsa-miR-661	
10351	85	hsa-miR-1237	*The target sequence is quite different in DENV4
10398	132	hsa-miR-943	
10558	292	hsa-miR-658	
10318	52	hsa-miR-658	
10319	53	hsa-miR-1908	*Different possition in DENV4
10555	289	hsa-miR-769-5p	
10360	94	hsa-miR-1268	
10557	291	hsa-miR-298	
10272	6	hsa-miR-370	
10530	264	hsa-miR-199a-5p	
10593	327	hsa-miR-937	
10365	99	hsa-miR-937	
10294	28	hsa-miR-1287	
10328	62	hsa-miR-623	
10470	204	hsa-miR-651	
10293	27	hsa-miR-302a-star	
10484	218	hsa-miR-522-star	
10293	27	hsa-miR-371-5p	
10462	196	hsa-miR-622	
10484	218	hsa-miR-518e-star	
10278	12	hsa-miR-373	
10484	218	hsa-miR-518f-star	
10547	281	hsa-miR-484	
10386	120	hsa-miR-195-star	
10319	53	hsa-miR-939	
10386	120	hsa-miR-16-1-star	
10403	137	hsa-miR-378-star	
10423	157	hsa-miR-361-5p	
10466	200	hsa-miR-520f	
10387	121	hsa-miR-770-5p	
10570	304	hsa-miR-1262	
10608	342	hsa-miR-886-5p	
10414	148	hsa-miR-30c-1-star	*Different possition
10278	12	hsa-miR-302a	
10387	121	hsa-miR-105	
10357	91	hsa-miR-324-5p	
10374	108	hsa-let-7a-2-star	
10484	218	hsa-miR-520c-5p	
10483	217	hsa-miR-554	
10392	126	hsa-miR-554	
10386	120	hsa-miR-16-2-star	
10349	83	hsa-miR-1274a	
10542	276	hsa-miR-330-5p	
10485	219	hsa-miR-330-5p	
10474	208	hsa-miR-139-5p	
10458	192	hsa-miR-1238	*Different possition
10495	229	hsa-miR-1238	
10404	138	hsa-miR-150	*Different possition in DENV2
10484	218	hsa-miR-526a	
10530	264	hsa-miR-199b-5p	
10467	201	hsa-miR-1911	
10511	245	hsa-miR-659	
10452	186	hsa-miR-659	
10289	23	hsa-miR-631	
10453	187	hsa-miR-1539	
10276	10	hsa-miR-550	
10344	78	hsa-miR-1225-5p	
10609	343	hsa-miR-1225-5p	
10589	323	hsa-miR-431	
10554	288	hsa-let-7c	
10362	96	hsa-miR-663b	
10276	10	hsa-miR-663b	
10484	218	hsa-miR-523-star	
10399	133	hsa-miR-345	
10457	191	hsa-miR-1274b	
10319	53	hsa-miR-654-5p	
10291	25	hsa-miR-1972	
10606	340	hsa-miR-15a	
10283	17	hsa-miR-564	
10484	218	hsa-miR-519b-5p	
10484	218	hsa-miR-519c-5p	
10485	219	hsa-miR-326	
10484	218	hsa-miR-519a-star	
10583	317	hsa-miR-1250	
10549	283	hsa-miR-744	
10318	52	hsa-miR-744	
		89	

Green: miRNAs common to serotypes 1 and 2
Yellow: miRNAs common to serotypes 1, 2 and 3
Blue: miRNAs common to serotypes 1, 2, 3 and 4
Red: miRNAs with more than one target within the genome of one serotype
Underlined: miRNAs common to the 4 serotypes and whose targets are in the same region of the genome (e.g. miR-637 has targets in all 4 serotypes, but the target in DENV4 is completely unrelated to the target in the genome of the other 3 serotypes[it is in a different localization])
*Each miRNA is counted only once, regardless of the number of target sites in the 3'UTR
