# Supplementary material for: Complex interaction between dengue virus replication and expression of miRNA-133a
Source: BMC Infect Dis. 2016 Jan 27;16:29. doi: 10.1186/s12879-016-1364-y (PMC4728791; doi:10.1186/s12879-016-1364-y)
Supplement: Supplementary file 2 — miRNA-133a predicted targets in the PTB1 gene. Predicted targets of MyomiRs are shown with the positions of target sequences in the 3′ UTR of mammalian mRNAs. (PDF 126 kb) [file 12879_2016_1364_MOESM2_ESM.pdf]

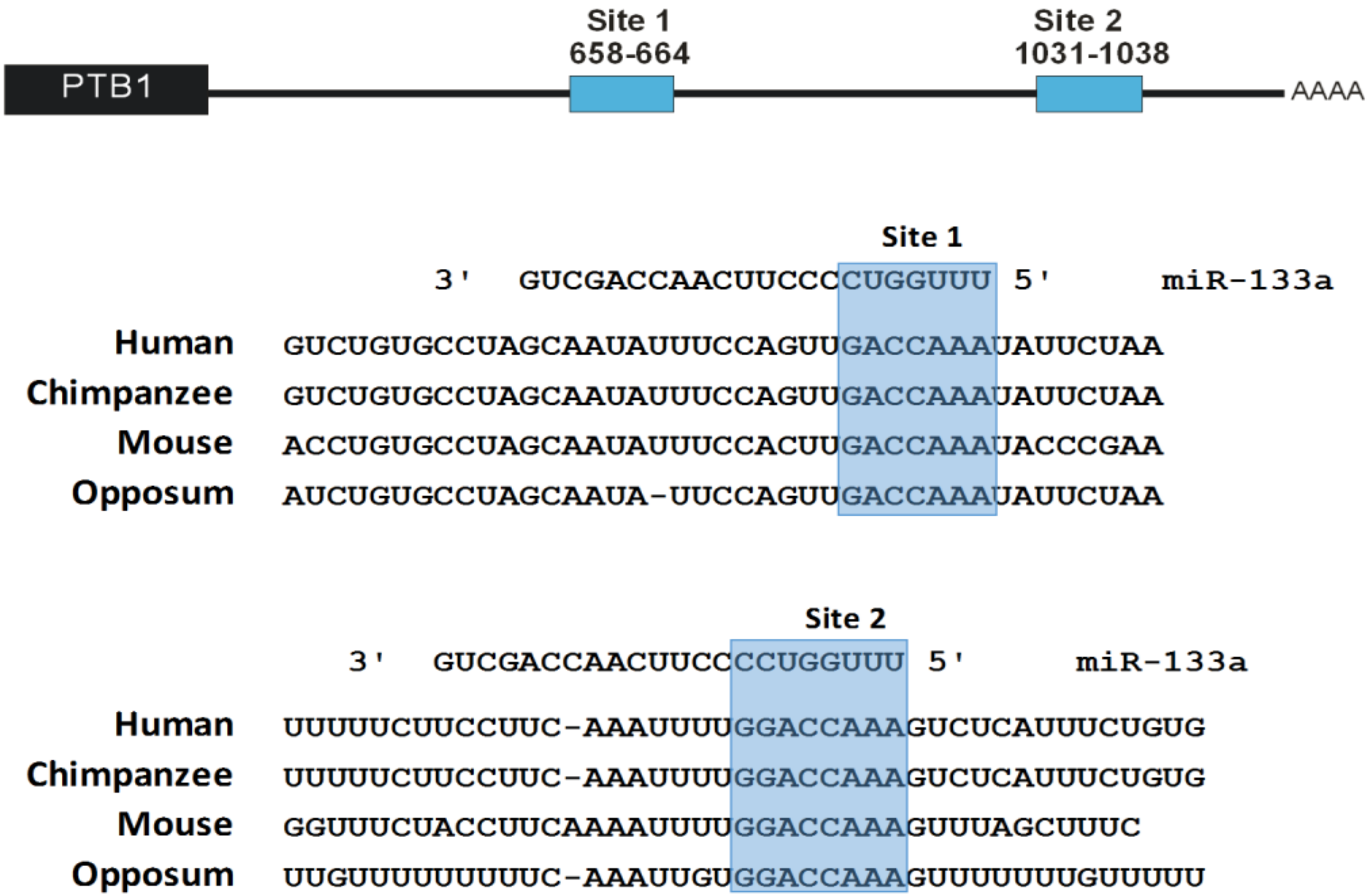

Site 1
658-664
Site 2
1031-1038
PTB1
AAAA
Site 1
3' GUCGACCAACUUCCCUGGUUU 5' miR-133a
Human GUCUGUGCCUAGCAAUAUUUCCAGUUGACCAAAUAUUCUAA
Chimpanzee GUCUGUGCCUAGCAAUAUUUCCAGUUGACCAAAUAUUCUAA
Mouse ACCUGUGCCUAGCAAUAUUUCCACUUGACCAAAUACCCGAA
Opposum AUCUGUGCCUAGCAAUA-UUCCAGUUGACCAAAUAUUCUAA
Site 2
3' GUCGACCAACUUCCCCUGGUUU 5' miR-133a
Human UUUUUCUUCCUUC-AAAUUUUGGACCAAAGUCUCAUUUCUGUG
Chimpanzee UUUUUCUUCCUUC-AAAUUUUGGACCAAAGUCUCAUUUCUGUG
Mouse GGUUUCUACCUUCAAAAUUUUGGACCAAAGUUUAGCUUUC
Opposum UUGUUUUUUUUUC-AAAUUGUGGACCAAAGUUUUUUUGUUUUU
