# Supplementary material for: Complex interaction between dengue virus replication and expression of miRNA-133a
Source: BMC Infect Dis. 2016 Jan 27;16:29. doi: 10.1186/s12879-016-1364-y (PMC4728791; doi:10.1186/s12879-016-1364-y)
Supplement: Supplementary file 3 — Endogenous expression of miR-133a in Vero cells infected with DENV-2. (DOCX 14 kb) [file 12879_2016_1364_MOESM3_ESM.docx]

|  | **Control** | | | | **DENV-2 infected** | | | |  |
| --- | --- | --- | --- | --- | --- | --- | --- | --- | --- |
|  | **Ct miR-133a** | **Ct 18S RNA** | **Normalized Ct** | **Efficiency** | **Ct miR-133a** | **Ct 18S RNA** | **Normalized Ct** | **Efficiency** | **Fold difference** |
| **8h** | 35,88 | 14,30 | 21,58 | 3,09E-06 | 37,67 | 13,35 | 24,31 | 6,19E-07 | **-4,99** |
| **12h** | 35,89 | 14,33 | 21,56 | 3,12E-06 | 39,85 | 13,53 | 26,31 | 1,91E-07 | **-16,33** |
| **24h** | 35,67 | 14,66 | 21,01 | 4,32E-06 | 37,19 | 13,81 | 23,37 | 1,07E-06 | **-4,01** |
| **36h** | 35,20 | 14,05 | 21,14 | 3,99E-06 | 34,54 | 13,90 | 20,63 | 5,38E-06 | 1,35 |
| **48h** | 36,20 | 14,22 | 21,98 | 2,44E-06 | 33,67 | 13,76 | 19,90 | 8,30E-06 | **3,40** |
| **72h** | 38,12 | 14,25 | 23,86 | 8,07E-07 | 34,89 | 14,04 | 20,84 | 4,76E-06 | **5,90** |
